# Meta-QTL Analysis in Rice and Cross-Genome Talk of the Genomic Regions Controlling Nitrogen Use Efficiency in Cereal Crops Revealing Phylogenetic Relationship

**DOI:** 10.3389/fgene.2021.807210

**Published:** 2021-12-21

**Authors:** Nitika Sandhu, Gomsie Pruthi, Om Prakash Raigar, Mohini Prabha Singh, Kanika Phagna, Aman Kumar, Mehak Sethi, Jasneet Singh, Pooja Ankush Ade, Dinesh Kumar Saini

**Affiliations:** ^1^ Punjab Agricultural University, Ludhiana, India; ^2^ Indian Institute of Science Education and Research, Berhampur, India

**Keywords:** candidate gene, grain yield, meta-QTL, NUE, orthologue, rice, root

## Abstract

The phenomenal increase in the use of nitrogenous fertilizers coupled with poor nitrogen use efficiency is among the most important threats to the environment, economic, and social health. During the last 2 decades, a number of genomic regions associated with nitrogen use efficiency (NUE) and related traits have been reported by different research groups, but none of the stable and major effect QTL have been utilized in the marker-assisted introgression/pyramiding program. Compiling the data available in the literature could be very useful in identifying stable and major effect genomic regions associated with the root and NUE-related trait improving the rice grain yield. In the present study, we performed meta-QTL analysis on 1,330 QTL from 29 studies published in the past 2 decades. A total of 76 MQTL with a stable effect over different genetic backgrounds and environments were identified. The significant reduction in the confidence interval of the MQTL compared to the initial QTL resulted in the identification of annotated and putative candidate genes related to the traits considered in the present study. A hot spot region associated with correlated traits on chr 1, 4, and 8 and candidate genes associated with nitrate transporters, nitrogen content, and ammonium uptake on chromosomes 2, 4, 6, and 8 have been identified. The identified MQTL, putative candidate genes, and their orthologues were validated on our previous studies conducted on rice and wheat. The research-based interventions such as improving nitrogen use efficiency *via* identification of major genomic regions and candidate genes can be a plausible, simple, and low-cost solution to address the challenges of the crop improvement program.

## Introduction

Asian rice (*Oryza sativa*) is a major cereal crop grown worldwide and an essential food source for over half of the world’s population (Nayar, 2014). The area under rice has continued to grow despite severe unsustainability issues due to national food security concerns and assured procurement on a minimum support price. Nitrogen is the essential macronutrient, and it is considered as the limiting factor for crop productivity. The nitrogen requirement of crops is highly dependent on the exogenous supply of nitrogen (Kraiser et al., 2011). The world’s agriculture is facing new challenges, and the global grain and food security problem persists (Kalugina, 2014). Globally, 119.41 million tons of nitrogen (N) fertilizers are being applied to the cereal crops to achieve the desirable crop yield (FAO, 2018). Rice needs nitrogen during whole stages of growth and development (Cassman et al., 1998; Ye et al., 2019). The rice crop has the lowest NUE among the major cereal crops (Norton et al., 2015). The increasing use of nitrogenous fertilizers for crop production coupled with poor nitrogen use efficiency (NUE) has led to the degradation of soil, water, and environment. The uptake, transport, assimilation/utilization, and remobilization of N are controlled by a complex and interconnected network of genes involved in various biological processes (Kant et al., 2011).

To date, a number of QTLs associated with nitrogen uptake, utilization, and nitrogen use efficiency have been detected in different cereal crop species (Agrama et al., 1999; Gallais and Hirel, 2004; Ribaut et al., 2007; Fontaine et al., 2009; Wei et al., 2012; Hu et al., 2015; Sandhu et al., 2015; Li et al., 2017; Zhou et al., 2017; Pozzo et al., 2018; Van Deynze et al., 2018; Zhang et al., 2019; Brasier et al., 2020). To the best of our knowledge, none of the identified genomic regions have been deployed in the marker-assisted introgression and pyramiding program. The QTL mapping approach generally influenced by the parents is used to develop the mapping population, choice of marker sets, population size and types, and the testing environments (Li et al., 2013; Lei et al., 2018; Zhao et al., 2018). Integrating QTL results from different independent experiments performed on the related cereal crop species provide useful information to study the genetic diversity of loci/alleles underlying the quantitative traits and highlight the potential targets to be further used in molecular breeding or the QTL cloning program.

The efficiency of any genomics-assisted breeding (GAB) program depends upon the consistency of the QTL effect across different genetic backgrounds and multiple environments (Collard and Mackill, 2008). The major effect QTLs which have been consistently reported in various studies in a common genomic region of the chromosome pinpoint its major role in regulating the particular trait to be further used efficiently in the GAB program.

Meta-QTL (MQTL) analysis is the approach to compile the information regarding consensus QTLs for a particular trait and to validate their effect across environments and backgrounds (Goffinet, 2000). Several studies have already been conducted to successfully locate the regions (meta-QTLs) in the genome of multiple crops for multiple traits (Li et al., 2013; Semagn et al., 2013; Acuña-Galindo et al., 2015; Van and McHale, 2017; Zhang et al., 2017; Khahani et al., 2021). The additional advantage of MQTL is the reduction of the confidence interval (CI) of the MQTLs controlling a particular trait of interest and identification of targeted candidate genes, thus improving the genetic resolution of the marker-assisted breeding program (Khahani et al., 2019). So, keeping in line the usefulness of meta-QTL analysis for narrowing down the major target genes, the present study is planned to aggregate all the reported QTLs for nitrogen use efficiency (NUE) and related traits and to fetch out relevant meta-QTL which can be further used in the breeding program with more precise knowledge about the genomic regions that can be targeted for NUE-associated traits in rice.

## Materials and Methods

The MQTL analysis for the nitrogen use efficiency (NUE) and related traits in rice was performed in the present study. It involves three main steps in identifying consensus QTLs associated with NUE and their related traits in rice:

1. An extensive bibliographic search of the QTL mapping studies and compilation of the reliable data on QTLs associated with NUE and related traits.
2. Creation of a consensus map on which QTLs of the individual studies were projected.
3. Meta-analysis on the QTL clusters to identify the consensus MQTL.

### Bibliographic Search and Data Mining

An extensive bibliographic search on the QTLs associated with the traits controlling NUE and related traits in rice was conducted and retrieved from Google Scholar, PubMed (http://www.ncbi.nlm. nih. gov/pubmed), and Mendeley using appropriate keywords such as the agro-morphological trait, developmental stage, grain yield, nitrogen application, nitrogen use efficiency, nitrogen deficiency, nitrogen uptake, nitrogen utilization, nitrogen remobilization, QTL, root trait, and rice. The literature reports published in journal articles and dissertations from 2000 to 2021 were considered, and all information on QTLs pertaining to nitrogen use efficiency (NUE) and its related traits was compiled for carrying forward the genome-wide meta-QTL analysis. QTL data including details on the parents used to develop the mapping population, type of mapping population (F_2_; recombinant inbred lines (RILs); backcross population (BC); near isogenic lines (NILs); chromosome segment substitution lines (CSSLs); double haploids (DHs)), size of population assayed, logarithm of the odds (LOD) score, phenotypic variance (*R* ^2^ or PVE), molecular markers flanking the QTL along with its genetic position (QTL position), and the genetic position of the QTL interval (confidence interval; CI) were compiled. The mid-point position between the two flanking markers was considered as the peak position wherever the information about the peak position was missing. The actual LOD score reported in the study was used, and in case, when the actual LOD score for an individual QTL was not available, a threshold LOD score of 3.0 was chosen for the study. The different QTLs located on the same chromosome were distinguished using the numerical identifiers following the chromosome. After careful examination, 28 relevant studies including 11-BC, 13-RILs, 3-DH, and 1-CSSL with all complete information from the 200 studies searched were compiled (Table 1). The studies that lacked the required data, such as the genetic position, LOD, and phenotypic variance, were excluded from the analysis. The size of the mapping populations ranged from 75 to 611 for BC lines, 101 to 291 for RILs, 113 to 233 for DH, and 247 for CSSL lines. The information on the 33 traits associated with NUE and its related traits was compiled for carrying forward the genome-wide meta-QTL analysis. The 33 traits were accommodated into 11 major trait categories (Table 2). The detailed information on 1,330 QTLs associated with 11 major trait categories including the agricultural nitrogen-absorption efficiency (ANAE)((65 QTLs)); nitrogen content (NC)(118 QTLs); nitrogen use efficiency (NUE)(73 QTLs); photosynthetic rate and chlorophyll content (SPAD) (60 QTLs); nitrogen-related enzymes and amino acids (NEAA) [proline (PRO), free amino acid (FAA), soluble proteins (SP), peroxidases (POD), nitrate reductase (NR), catalase (CAT), glutamine synthetase (GSI), NADH-glutamate synthetase (NGOC)] (53 QTLs); root traits (RT) [root length (RL), root number (RN), root thickness (RT), root dry weight (RDW)] (53 QTLs); plant growth and morphological traits such as plant height (PH) (105 QTLs); shoot dry weight (SDW) (57 QTLs); total dry biomass (TDB) (89 QTLs); leaf number and area (LN and A) (20 QTLs); and grain yield (GY) and yield-related traits [biomass yield (BY), grain weight (GW), harvest index (HI), days to 50% flowering (DTF), tiller number (TN), panicle length (PL), panicle number per plant (PNP), number of grains per panicle (NGP), spikelet fertility (SF), partial factor productivity (PFP)](630 QTLs) were compiled. The compiled information on the 1,330 QTLs associated with the 11 trait categories was subjected to MQTL analysis. The QTLs have been renumbered based on their location on each chromosome.

**TABLE 1:** Summary of the QTL studies used in the QTL meta-analysis for the nitrogen use efficiency and related traits in rice.

Cross	Cross type	Pop size	No of markers	Types of markers	Total no of QTL	Traits (QTL) identified per traits	Year of study	Country	References
Habataki × Koshihikari	BC_5_F_3_/F_4_	611	23	SSR	2	Photosynthetic rate (PS) (2)	2011	Japan	Adachi et al. (2011)
XieqingzaoB × Zhonghui9308	BC_4_F_6_-CSSL	75	55	SSR	9	plant height (PH) (3), shoot dry weight (SDW)(2), root dry weight (RDW)(1), total dry biomass (TDB)(1), root length (RL)(1), root number (RN)(1)	2016–2017	China	Anis et al. (2018)
CSSL45× Zhonghui9308	BC_5_F_2_/F_3_	75	10	SSR	1	root dry weight (RDW) (1)	2017	China	Anis et al. (2019)
XieqingzaoB × Zhonghui_9308	RIL	281	118	SSR	13	nitrogen content (NC)(4), harvest index (HI)(2), nitrogen use efficiency (NUE)(2), agricultural nitrogen-absorption efficiency (ANAE)(5)	2008–2009	China	Dai et al. (2015)
ZS97 × MH63	RIL	127	141	SSR, RFLP	30	grain yield (GY)(9), biomass yield (BY)(6), nitrogen content (NC)(8), nitrogen use efficiency (NUE)(7)	2006–2007	China	Wei et al. (2012)
ZS97 × MH63	RIL	127	115	SSR, RFLP	68	number of grains per panicle (NGP)(10), grain yield (GY)(14), panicle number per plant (PNP)(12), spikelet fertility (SF)(10), grain weight (GW)(22)	2006–2007	China	Wei et al. (2012)
IR64 × Azucena	DH	123	113	RFLP, RAPD	18	nitrogen content (NC)(6), tillering number (TN)(4), days to 50% flowering (DTF)(18)	2000	China	Fang and Wu, (2001)
R9308 × XieqingzaoB	RIL	238	82	SSR	7	shoot dry weight (SDW)(3), total dry biomass (TDB)(2), root length (RL)(1), plant height (PH)(1)	2008	China	Feng et al. (2010)
XQZB × R9308	RIL	138	127	SSR	21	plant height (PH)(11), days to 50% flowering (DTF)(10)	2009	China	Feng et al. (2011)
Lemont × Teqing	CSSL	247	118	SSR, RAPD	31	plant height (PH)(8), number of panicles per plant (PNP)(7), chlorophyll content (SPAD)(8), shoot dry weight (SDW)(5), grain yield (GY)(3)	2003	China	Han-Hua et al., 2006
WTR-1 × HAN	BC_1_F_5_	230	98	SNP	261	grain yield (GY)(50), nitrogen use efficiency (NUE)(4), biomass yield (BY)(40), partial factor productivity (PFP)(22), grain weight (GW)(61), spikelet fertility (SF)(84)	2014	Philippines	Jewel et al. (2019)
WTR1 × HAN_CH448_Z413	BC_1_F_5_	243	38	SNP	19	chlorophyll content (SPAD)(5), plant height (PH)(5), tiller number (TN)(9)	2017	Philippines	Mahender et al. (2019)
US-2 × Malay-2	BC_1_F_4_-RIL	168	83	SSR	8	root length (RL)(2), root number (RN)(3), root dry weight (RDW)(2), plant height (PH)(1)	2007	Philippines	Manangkil et al. (2019)
IR64 × Azucena	DH	84	135	RFLP, RAPD	16	days to 50% flowering (DTF)(1), number of grains/panicle (NGP)(2), nitrogen content (NC)(2), nitrogen use efficiency (NUE)(1), plant height (PH)(3), panicle length (PL)(1), tiller number (TN)(4), grain yield (GY)(1), spikelet fertility (SF)(1)	2008	Philippines	Senthilvel et al. (2008)
BPT5204 × PTB1	RIL	291	25	SSR	37	days to 50% flowering (DTF)(8), number of grains per panicle (NGP)(1), grain yield (GY)(2), leaf length (LL)(4), plant height (PH)(5), biomass yield (BY)(1), nitrogen content (NC)(8), chlorophyll content (SPAD)(2), total dry biomass (TDB)(1), grain weight (GW)(5)	2014–2015	India	Vishnukiran et al. (2020)
Lijiangxintu-anheigu × Towada	BC_4_F_10_/F_11_	105	94	SSR	47	soluble protein (SP)(17), free amino acid (FAA)(5), proline (PRO)(6), catalase (CAT)(7), peroxidase (POD)(5), nitrate reductase (NR)(7)	2017	China	Yang et al. (2019)
XieqingzaoB × Zhonghui9308	RIL	138	165	SSR	52	panicle length (PL)(10), spikelet fertility (SF)(3), number of panicles per plant (PNP)(20), grain yield (GY)(3), number of grains per panicle (NGP)(13), grain weight (GW)(3)	2009	China	Yue et al. (2015)
*indica*cultivar9311× *japonica* Nipponbare	BC_4_F_2_	119	190	SSR	44	plant height (PH)(9), root length (RL)(5), root dry weight (RDW)(2), shoot dry weight (SDW)(17), total dry biomass (TDB)(11)	2012	China	Zhao et al. (2014)
IR64 × INRC10192	RIL	140	60	ISSR	46	total dry biomass (TDB)(1), number of grains per panicle (NGP)(5), spikelet fertility (SF)(6), grain weight (GW)(1), grain yield (GY)(3), harvest index (HI)(9), number of tillers (NT)(5), plant height (PH)(15), panicle length (PL)(1)	2005	India	Akkareddy et al. (2010)
IR64 × Azucena	RIL	174	228	SSR	446	nitrogen content (NC)(63), agricultural nitrogen-absorption efficiency (ANAE)(50), nitrogen use efficiency (NUE)(56), biomass yield (BY)(1), grain weight (GW)(3), grain yield (GY)(5), leaf area (LA)(1), number of grains per panicle (NGP)(30), number of leaves (NL)(9), plant height (PH)(23), panicle number per plant (PNP)(26), photosynthetic rate (PS)(17), root dry weight (RDW)(19), shoot dry weight (SDW)(30), chlorophyll content (SPAD)(23), total dry biomass (TDB)(65), total fresh weight (TFW)(5), tiller number (TN)(20)	2011	Belgium	Nguyen et al. (2014)
Dasanbyeo × TR22183	RIL	166	6	SSR, STS	5	nitrogen content (NC)(1), harvest index (HI)(1), grain yield (GY)(1), biomass yield (BY)(2)	2002	Korea	Cho et al. (2007)
ZYQ8XJ × 17	DH	127	233	SSR	28	plant height (PH)(15), tiller number (TN)(13)	2003	China	Jiang et al. (2008)
Nipponbare × kasalath	BC_1_F_6_	98	45	RFLP	12	nitrogen content (NC)(6), NADH-glutamate synthetase content (NGOC)(6)	2001	Japan	Obara et al. (2014)
YTH183 × IR64	BC_3_F_8_	334	17	SSR	5	root length (RL)(5)	2010–2011	Japan	Obara et al. (2014)
NPT × IR72	RIL	101	170	SSR, RFLP	61	days to 50% flowering (DTF)(7), grain weight (GW)(3), spikelet fertility (SF)(3), nitrogen content (NC)(20), nitrogen use efficiency (NUE)(3), plant height (PH)(3), panicle number per plant (PNP)(4), specific leaf area (SLA)(6), chlorophyll content (SPAD)(3), total dry biomass (TDB)(4)	2001–2004	Japan	Laza et al. (2006)
HHZ × Teqing, CDR22, OM1723	BC_1_F_4_	206	4	KASP SNP	2	grain yield (GY)(1), spikelet fertility (SF)(1)	2014	China	Feng et al. (2018)
Azucena × Bala	RIL	205	65	SSR	17	root length (RL)(2), maximum root thickness (MRT)(2), root dry weight (RDW)(6), total dry biomass (TDB)(4), plant height (PH)(3)	2009	United Kingdom	MacMillan et al. (2006)
Zhenshan 97 × Minghui 63	RIL	127	108	SSR	24	agricultural nitrogen-absorption efficiency (ANAE)(10), grain yield (GY)(14)	2006–2007	China	Wei et al. (2011)

BC: backcross, RIL: recombinant inbred lines, DH: double haploids, CSSL: chromosome segment substitution lines, RAPD: random amplified polymorphic DNA, RFLP: restriction fragment length polymorphism, SSR: simple sequence repeats, ISSR: inter-simple sequence repeats, SNP: single nucleotide polymorphism, KASP: kompetitive allele specific PCR, STS: sequence-tagged sites. The numeric number in the bracket () represents the number of QTL, for that particular trait.

**TABLE 2:** The trait category and number of QTL compiled per category in the QTL meta-analysis for the nitrogen use efficiency and related traits in rice.

Trait category	Number of QTL
Agricultural nitrogen-absorption efficiency	65
Nitrogen content	118
Nitrogen use efficiency	73
Nitrogen-related enzymes and amino acids (proline, peroxidases, nitrate reductase, free amino acid, soluble proteins, catalase, NADH-glutamate synthetase)	53
Photosynthetic rate and chlorophyll content	60
Plant height	105
Shoot dry weight	57
Total dry biomass	89
Leaf number and area	20
Root traits (root length, root number, root thickness, root dry weight)	53
Grain yield and related traits (harvest index, biomass yield, number of grains per panicle, panicle length, panicle number per plant, grain weight, days to 50% flowering, number of productive tillers, spikelet fertility, partial factor productivity)	637
Total	1,330

### QTL Projection and Development of the Consensus Map

To construct a consensus map, the LPmerge tool in the R package was employed (Endelman and Plomion, 2014) where the maps with the markers and QTLs were iteratively projected on a composite reference map (integrated rice genetic linkage maps). The reference maps used were those from Orjuela et al. (2010), Supplementary table 18 from GRAMENE (https://archive.gramene.org (International Rice Genome Sequencing Project, 2005), Cornell SSR 2001, and IRMI-2003 (https://archive.gramene.org). It is based on the common marker between the original map and reference map by means of a homothetic function described by Chardon et al. (2004). Any marker that did not comply (inverted) in terms of linkage was automatically discarded. After the integration of all the maps, the consensus map contained 21,280 markers, including SSR, RFLP, AFLP, SNP markers, and genes. The total number of markers discarded was 560 markers. The consensus map covered a total length of 1821 cM, with an average distance of 3.5 cM between markers. Only 1,330 QTLs from the 2,763 available QTLs associated with the 11 trait categories with complete information (estimated CIs, original LOD scores, peak positions, and phenotypic variance) required for the QTL projection were used for projection on the consensus map using BioMercator V4.2 (Sosnowski et al., 2012) (Table 2).

### Meta-QTL Analysis

Following projection, the meta-QTL analysis was performed, for each chromosome individually following the Veyrieras et al. (2007) two-step algorithm available in the software BioMercator V4.2. The Akaike (AIC) statistics values were used to find the best QTL model for determining the actual number of MQTLs on each of the rice chromosome. The detailed statistical procedures and the algorithms used in this software have been well-described in the study by Sosnowski et al. (2012). The first step involved the use of five different models based on the presence of 1, 2, 3, 4, or N real QTL. The best model was selected using Akaike (AIC) statistics. The second step involved setting up the suitable parameters for further analysis. The parameters used were the actual number of MQTLs or the real QTL to be mapped on the concerned chromosome. The phenotypic variance and LOD values of the MQTLs were calculated as the averages of the phenotypic variance and LOD values of the QTLs involved.

### Functional Analysis and Identification of Nitrogen Use Efficiency–Associated Candidate Genes

The selected 42 meta-QTL regions possessing QTLs with average phenotypic variance >8%, average LOD >4, and the involvement of ≥10 initial QTLs within the MQTL were subjected to the functional analysis to identify candidate genes related to the traits associated with nitrogen use efficiency in rice. First, genetic markers flanking the confidence intervals of each rice MQTL (rMQTL) were selected, and their physical positions on respective chromosomes were obtained from the Gramene database (www.gramene.org), or gene models present within the original or estimated physical regions were retrieved using the “BioMart” of the Ensembl Plants database. All the genes physically located within or near each rice MQTL region were considered as candidate genes and retrieved from the Rice Annotation Project Database (RAPDB) (http://rapdb.dna.affrc.go.jp) as batch download, or the primer sequence of the marker is subjected to nucleotide blast in NCBI (https://blast.ncbi.nlm.nih.gov/Blast.cgi?PROGRAM=blastn) to identify the range of the sequence in the reference genome of Nipponbare using RAP and Build 5 (www.rapdatabase.org). It is assumed that the genes identified in Nipponbare regions are homologous and collinear to those underlying the nitrogen use efficiency and associated QTLs mapped in different studies involving different donor wild species and recipients. The functional annotations of the identified gene models were explored for the best candidate genes within each MQTL. All genes with the gene ontology (GO) term or description related to NUE, nitrogen uptake, its assimilation, and all related traits were filtered and considered as candidate genes (CGs). The QTLs associated with nitrogen use efficiency and related traits identified previously by Sandhu et al. (2015), Sandhu et al. (2016), Sandhu et al. (2019), Subedi et al. (2019), and Sandhu et al. (2021) were projected on the MQTLs identified in the present study to find consistency in the genomic regions.

### Rice MQTL Regions Homologous to the Other Cereals

Additionally, to evaluate transferability of information to other cereals, ortho-MQTLs were investigated based on the genomic collinearity between rice–maize, rice–wheat, and rice–barley. The information on maize, wheat, and barley genes associated with NUE and related traits was collected from the available literature and used for the retrieval of corresponding protein sequences for the identification of homologous MQTL genomic regions. The amino acid sequences for the relevant genes were then retrieved from NCBI https://www.ncbi.nlm.nih.gov/) and used for BLASTP search to identify the rice protein (available in Ensembl Plants) at an E-value of <10^–10^, with 60% coverage, and >60% identity. The physical positions of the corresponding genes and rice MQTLs were then compared to detect the MQTL regions homologous to known genes in other cereals.

To detect the ortho-MQTLs between rice and maize, rice and wheat, and rice and barley syntenic regions between the two respective species were identified by using the Ensembl Plants database.

The distribution of the aforementioned factors, number of MQTL related to nitrogen use efficiency and all related traits, and candidate genes underlying MQTL over the rice genome were shown by using Circos (Krzywinski et al., 2009).

### Development of the Validation Panel to Check the Efficacy of the Identified MQTL and Candidate Genes.

The information on the marker trait associated with root traits improving nitrogen use efficiency and grain yield/yield-related traits in rice (Sandhu et al., 2015; Sandhu et al., 2019; Subedi et al., 2019) and wheat (Kumar et al., 2021; Sandhu et al., 2021) was collected from our previous studies. The information on the nitrate transporter gene in wheat and rice was collected from the Ensembl Plants database. To check the nitrogen responsiveness of candidate genes, the expression data of all the candidate genes were downloaded from the RiceXPro database. Specifically, root gene expression profiles in response to nitrogen were considered. The dataset RXP_5002, which contained microarray-based expression data of 7-day-old seedlings exposed to nitrogen deficiency treatments and control conditions, was analyzed.

## Results

### Salient Features of the QTL Studies

**FIGURE 1:**
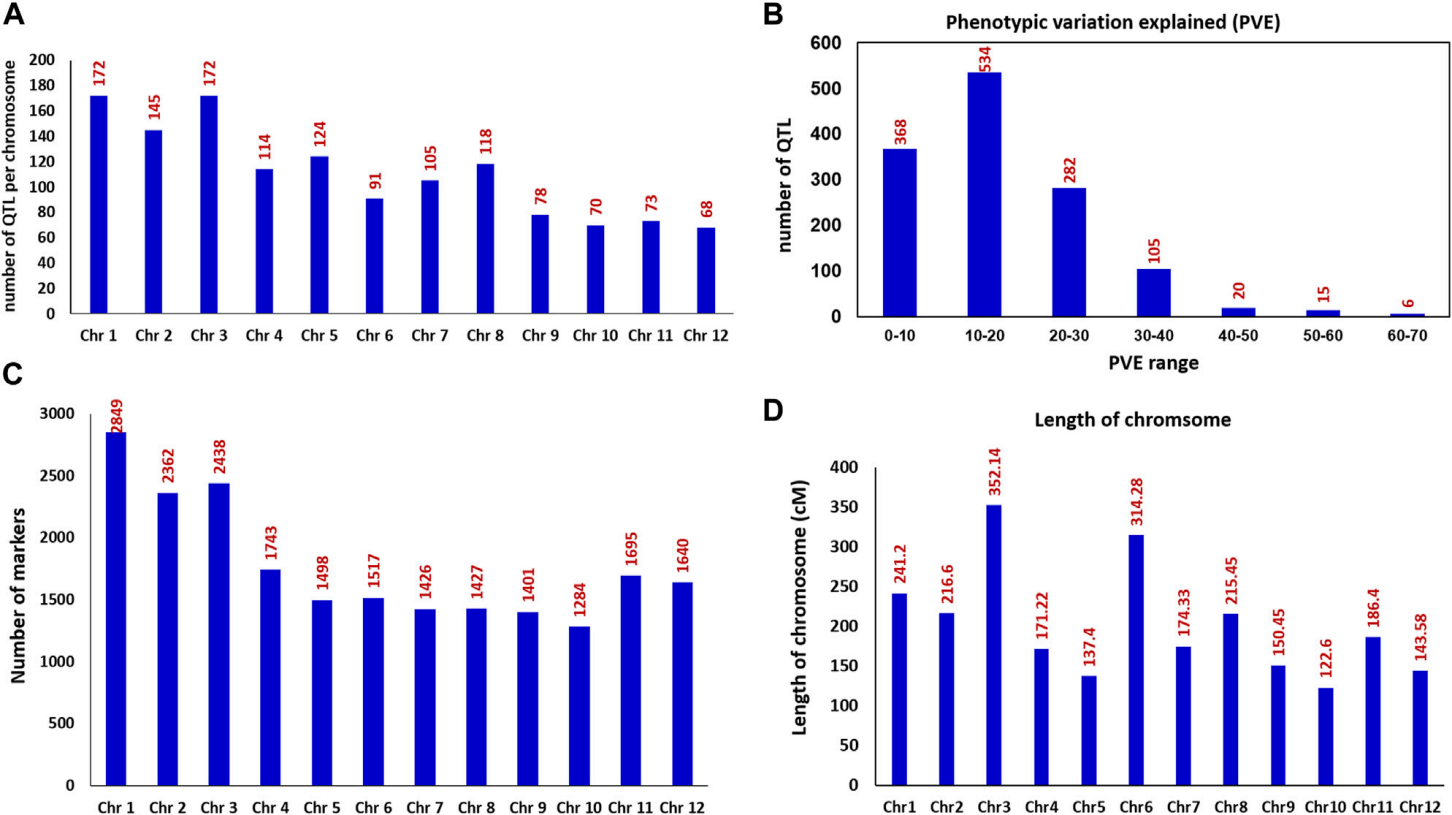
Frequencies of QTL and details on the mapped markers used for the identification of MQTLs in rice **(A)** Number of QTLs present on the 12 rice chromosomes **(B)** Frequencies of the QTL with different levels of phenotypic variation explained (PVE%) **(C)** Frequencies of the mapped markers on each of the 12 rice chromosomes **(D)** Length/size of the rice genetic map in cM (centimorgan) across 12 chromosomes.

In order to identify the consensus genomic regions associated with the 33 nitrogen use efficiency and related traits, we compiled the information on a total of 1,330 QTLs derived from 28 relevant studies including 11-BC, 13-RILs, 3-DH, and 1-CSSL with the population size that ranged from 75 to 611 reported between 2001 and 2021. All the 33 traits were grouped into 11 main trait categories (Table 2). The 1,330 QTLs were distributed across all 12 chromosomes (chr) of rice (Figure 1A). The chr 1 and 3 harbored the maximum number of QTL (172 QTL) for all the studied traits followed by chr 2 (145 QTL) and chr 5 (126 QTL). The chr 12 harbored the lowest number of QTL (68 QTL). Among the studied trait category, GY and related trait categories had the highest number of QTL with 630 QTLs followed by 118 QTLs for nitrogen content and 105 QTLs for plant height. The phenotypic variance of the individual QTLs ranged from 1.9 to 66.9%, with an average of 17.43%. The frequency distribution of the QTL with different levels of phenotypic variation explained is presented in Figure 1B. The LOD value of the individual QTL ranged from 2.02 to 28.7 with an average of 4.77.

### Rice Consensus Map

The rice consensus genetic map included a total of 22,280 markers (Figure 1C) covering a total length of 2425.65 cM, thus giving a density of 8.77 markers/cM for the whole rice genome. The size of the rice genetic map for an individual chr ranged from 122.60 cM (chr 10) to 352.14 cM (chr 3) (Figure 1D), and the number of markers on an individual chr ranged from 1,284 on chr 10 to 2849 on chr 1. The marker density on individual chr varied from 4.83 markers/cM for chr 6 to 11.81 markers/cM for chr 1.

### MQTLs Detected for Grain Yield/Related Traits and Nitrogen Content/Nitrogen Use Efficiency

Only 915 QTLs from the 1,330 available QTLs could be used for successful projection onto the consensus map; the remaining 415 QTLs could not be projected as either the associated markers were absent in the consensus map, or they had comparatively low PVE values and/or a large confidence interval. The MQTL analysis significantly summarized the total number of projected QTLs from 915 to 76 MQTLs (8.3%) (Table 3). The number of MQTLs per rice chromosome ranged from three (chr 1) to nine (chr 3) with an average of 6.33 MQTLs per chromosome. The 76 MQTLs involving 636 QTLs were identified using the MQTL analysis. The remaining 279 QTLs could not be assigned to any of the identified MQTL since they either lacked the common markers between the consensus map and initial maps or the QTL had a relatively low phenotypic variance or LOD value and/or a large confidence interval.

**TABLE 3:** Summary of the detected MQTLs for the nitrogen use efficiency and related traits in rice.

MQTL ID	Chr	Position (cM)	CI (95%)	From	To	Flanking marker from	Flanking marker to	Start (Mb)	End (Mb)	No. of QTLs involved in MQTL	QTLs involved
*MQTL1.1*	1	54.16	1.16	53.16	54.32	RM10009	RM10111	188501	2104274	47	SF, BY, GW, PNP, GD, GY, POD, PNP, RDW, TN, CAT, DTF, NC, PL, NUE, SP, SPAD, NGP, PFP, PH, HI
*MQTL1.2*	1	79.92	1.54	78.92	80.46	RM10523, SNP_1_20238919	RM10527, SNP_1_20706894	20238919	20706894	10	PH, GW, PNP, NGOC, DTF, NC, BY, SF, GY
*MQTL1.3*	1	110.58	0.12	109.58	109.7	RM11226	RM11235	22330477	22473962	26	BY, SPD, GY, PH, SDW, DTF, TDB, ANUE, NC, RT, PS, RL, PNP, SF, TN
*MQTL2.1*	2	16.71	2.96	15.23	18.19	SNP_2_4342883	SNP_2_4481943	4342883	4481943	16	GY, HI, BY, GW, PFP, SF
*MQTL2.2*	2	28.81	1.47	28.08	29.55	RM12349	RM12353	977799	1078287	2	NUE, PNP
*MQTL2.3*	2	39.76	2.14	38.69	40.83	RM12446	RM12461	2331733	2675901	13	PH, SF, NC, PNP, GW, ANAE, PL, GY, ANUE
*MQTL2.4*	2	51.72	1.58	50.93	52.51	RM12658	RM12693	5366392	6121755	11	DTF, SF, ANUE, TDB, GY, PNP, TN, PH
*MQTL2.5*	2	68.63	0.28	68.49	68.77	RM12913	RM12914	9243319	9409837	2	NC, DTF
*MQTL2.6*	2	69.67	1.09	69.13	70.22	RM12914	RM12918	9409837	9459499	5	DTF, NC, NGP
*MQTL2.7*	2	87.85	4.47	85.62	90.09	S2-136	G1314A	1291679	25433673	6	GY, SF, NC, GW
*MQTL2.8*	2	119.49	0.01	119.49	119.50	C747	G57	27078652	28307630	5	PNP, NC, DTF, PH
*MQTL3.1*	3	3.67	2.46	2.44	4.90	SNP_3_1270943	SNP_3_1670761	1270943	1670761	19	GW, SF, GY, BY, PFP, PH
*MQTL3.2*	3	14.49	4.68	12.15	16.83	SNP_3_3542519	RM14441	3542519	3597356	5	GY, SF, PFP, HI
*MQTL3.3*	3	49.05	1.44	48.33	49.77	RM4992	RM14526	4706112	5024749	12	RDW, SDW, PS, NUE, ANAE, SF, NT, TDB
*MQTL3.4*	3	65.42	9.43	60.71	70.14	SNP_3_16294363	RM15181	16294363	16310268	3	GY, DTF, GW
*MQTL3.5*	3	77.1	3.01	75.60	78.61	C6	RM545	2985072	4916484	11	PNP, NUE, GY, FAA, TN, GW, NC, RL
*MQTL3.6*	3	85.08	2.49	83.84	86.33	RM14782	RM14820	10140286	10790118	2	SDW, PH
*MQTL3.7*	3	95.69	0.49	95.45	95.94	RM14936	RM14945	12917488	12998073	10	DTF, SF, GY, PRO, PNP, POD
*MQTL3.8*	3	117.9	1.61	117.10	118.71	RM15288	RM15313	18945114	20112723	9	TN, PNP, NC, DTF, GY, SF
*MQTL3.9*	3	138.2	0.45	137.98	138.43	RM15626	RM15639	25803162	26022473	5	PHT, PL, SF, NUE, NC
*MQTL4.1*	4	4.37	1.18	3.78	4.96	RM16393	RM16399	3393833	3498871	6	ANAE, NC, DTF, SF, PS
*MQTL4.2*	4	32.74	3.17	31.16	34.33	RM16767	RM16788	17456500	18062531	4	NUE, TN, SF, NC
*MQTL4.3*	4	46.99	3.16	45.41	48.57	SNP_4_14609247	RM16926	14609247	18062531	10	SF, GW, PHT, TN, RDW, PNP, NC
*MQTL4.4*	4	59.1	2.39	57.91	60.30	RM16996	RM17007	21359732	21484908	21	NC, BY, GW, SF, PFP, GY, TDB, RDW, SDW, NUE, PNP, TN
*MQTL4.5*	4	72.97	0.98	72.48	73.46	SNP_4_21815986	SNP_4_21833014, RM17090	21815986	21833014	6	NGOC, PH, NGP, RL, TDB, GY
*MQTL4.6*	4	87.7	2.75	86.33	89.08	RM17231	RM17272	25746072	26405863	3	NC, GY, PNP
*MQTL4.7*	4	104.9	1.13	104.34	105.47	RM17475	RM17489	31082124	31418566	3	DTF, PH, SF
*MQTL5.1*	5	6.73	0.78	6.34	7.12	RM17846	RM17852	1777550	1942400	2	SF, DTF
*MQTL5.2*	5	18.02	2.52	16.76	19.28	GA478	R2232	2011188	4107103	20	PHT, PNP, SF, GY, BY, GW, NUE, SDW, PFP, NC
*MQTL5.3*	5	35.88	6.38	32.69	39.07	RM18408	SNP_5_15469279	15116392	15469279	2	TN, GY
*MQTL5.4*	5	44.56	3.03	43.05	46.08	RM18033	RM18071	5273339	6155982	9	PS, RL, PFP, GY, BY
*MQTL5.5*	5	51.56	3.09	50.02	53.11	RM18115	RM18176	7121001	8674028	5	RN, SF, SPD, NC, TDB
*MQTL5.6*	5	60.33	2.79	58.94	61.73	RM18302	RM18343	12708022	13572971	7	TDB, SF, RDW, ANAE, SDW
*MQTL5.7*	5	70.03	0.6	69.73	70.33	RM18624	RM18632	19183516	19346291	10	PH, RT, RL, TN, PNP, RDW, TN
*MQTL5.8*	5	110.22	0	110.22	110.22	RM178	RM6972	25101829	25208346	4	SF, RL
*MQTL6.1*	6	7.27	7.24	3.65	10.89	RZ242	G342	28963386	30822714	2	SF
*MQTL6.2*	6	44.18	1.6	43.38	44.98	SNP_6_12183428	SNP_6_13250266	12183428	13250266	21	TN, PFP, NUE, GW, GY, SF, BY, NC, RDW
*MQTL6.3*	6	62.07	1.71	61.22	62.93	R2549	RZ516	24916395	2560318	9	PL, DTF, TDB, BY, RDW, NUE, PNP, SDW, ANAE
*MQTL6.4*	6	93	2.52	91.74	94.26	RM19715	RM19746	7868951	8518493	12	PH, CAT, PRO, NC, SP, FAA, PNP, SF
*MQTL6.5*	6	107.6	1.9	106.65	108.55	SNP_6_29056693	SNP_6_29416997	29056693	29416997	14	GY, SF, TDB, TN, RN, BY, PNP, RL
*MQTL6.6*	6	159.12	1.43	158.41	159.84	Pho2	G329	27384548	27612443	6	NC, RL, DTF, SDW, GY
*MQTL7.1*	7	13.77	5.57	10.99	16.56	SNP_7_4569035	SNP_7_5704192	4569035	5704192	4	SF, BY
*MQTL7.2*	7	38.56	0.94	38.09	39.03	RM21034	RM21050	3577411	3771121	17	PNP, SF, PH, PL, BY, DTF, RL, POD, HI, SP, GW
*MQTL7.3*	7	48.82	2.24	47.70	49.94	RM21660	RM22027	18996018	26301017	13	NC, SP, GY, PRO, TDB, PS, TN, SDW, BY, DTF
*MQTL7.4*	7	80.47	2.6	79.17	81.77	RM22132	RM22157	28665611	29170514	12	GY, PNP, NUE
*MQTL7.5*	7	107.07	0.76	106.69	107.45	SNP_7_28234334	SNP_7_28303039	28234334	28303039	11	GW, SF, PFP, BY, GY
*MQTL8.1*	8	1.48	4.28	0.66	3.62	SNP_8_389278	G278	389278	1193267	5	PFP, GY, BY
*MQTL8.2*	8	18.73	2.35	17.56	19.91	RM6863	RM22416	2005990	3285143	9	BY, GW, NGP, HI, SF
*MQTL8.3*	8	31.49	3	29.99	32.99	SNP_8_8437588	SNP_8_8580913, RM122191	8437588	8580913	10	NC, BY, GW, SDW, PFP, GY
*MQTL8.4*	8	48.35	1.21	47.75	48.96	RM22335	RM22351	2144719	2470019	8	GY, DTF, PNP, SF, PL, PS
*MQTL8.5*	8	58.94	5.75	56.07	61.82	RM22981	S8_8206216, RM339	2470019	8206216, 17945059	2	SDW, PH
*MQTL8.6*	8	88.55	0.24	88.43	88.67	RM22979	RM22982	17250943	17401871	26	NC, TDB, GW, GY, SF, PH, NUE, RDW, ANAE, SPAD, TN, PNP
*MQTL9.1*	9	24.5	4.38	22.31	26.69	SNP_9_12154616	C397B	12154616	12289001	5	PH, GY, SF, BY
*MQTL9.2*	9	37.47	4.04	35.45	39.49	R1164	RZ698	6015994	7222547	10	GY, SF, BY, PFP, BY, GW
*MQTL9.3*	9	51.3	0.86	50.87	51.73	RM23654	RM23655	99249	159416	3	TDB, PNP, PS
*MQTL9.4*	9	56.73	3.49	54.99	58.48	RM23820	RM23888	5036985	6546861	12	ANAE, NC, TDB, GY, NUE, RDW, SF, GW
*MQTL9.5*	9	68.16	3.32	66.50	69.82	RM23967	RM23999	8127499	8970550	12	SDW, TDW, GW, NC, GY, RDW, PNP, SPAD, TN
*MQTL9.6*	9	86.82	0.49	86.58	87.07	RM24130	RM6839	11610977	14512398	6	GW, TN, PN, RDW, NC
*MQTL10.1*	10	10.33	4.71	7.98	12.69	SNP_10_2056123	RM24990	2056123	2768779	4	PH, ANAE, NC, SF
*MQTL10.2*	10	27.76	4.42	25.55	29.97	RM25084	RM25178	4818641	8255065	8	FAA, NC, NUE, TN, SDW, TDB
*MQTL10.3*	10	41.89	6.53	38.63	45.16	RM25271	S10_14563405	10748123	14563405	2	NC, PS
*MQTL10.4*	10	50.86	3.79	48.97	52.76	RM25308	RM25331	11746315	12461164	2	PNP, GY
*MQTL10.5*	10	56.87	3.37	55.19	58.56	RM467	RM25401	13044511	13797426	6	FAA, SP, PNP, TN
*MQTL10.6*	10	74.14	4.94	71.67	76.61	RM25601	SNP10_18820606	17570667	18820606	5	NC, GY, BY, TDB
*MQTL10.7*	10	88.16	4.24	86.04	90.28	RM25852	RM25934	21605343	22626576	2	PNP, NC
*MQTL11.1*	11	23.05	5.99	20.06	26.05	RM26044	RM26108	1885593	2828718	4	HI, NC, SDW, NC
*MQTL11.2*	11	44.9	2.33	43.74	46.07	RM26306	RM26341	7073686	7650323	9	TDB, TN, NC, PNP, TDB, ANAE, SF
*MQTL11.3*	11	63.45	3.05	61.93	64.98	RM26687	RM26727	15903868	16610716	4	GY, NUE, PH
*MQTL11.4*	11	99.77	3.13	98.21	101.34	RM27045	RM27097	22663165	23487875	5	NR, RDW, ANUE, GY
*MQTL12.1*	12	14.75	5.57	11.97	17.54	RM27494	S12_5905028	1709959	5905028	3	TN, PNP, PH
*MQTL12.2*	12	49.07	6	46.07	52.07	RM28004	RM28064	1,3161862	14701301	2	PNP, HI
*MQTL12.3*	12	57.06	2.36	55.88	58.24	RM28095	RM28117	15679490	16360229	3	NUE, PNP
*MQTL12.4*	12	66.78	2.39	65.59	67.98	RM27712	RM27800	5104402	7237077	8	NC, PS, TDB, ANAE, SPAD, SDW, TDW, RDW
*MQTL12.5*	12	74.49	4.54	72.22	76.76	RM27855	RM28004	8458858	1,3161862	2	NC, DTF
*MQTL12.6*	12	86.42	5.01	83.92	88.93	RM28093	RM28455	15616573	22776128	2	PH
*MQTL12.7*	12	116.74	0.77	116.36	117.13	RM28511	RM28523	23594375	23753217	5	PNP, GY, TN, NGP, NC

ANAE: agricultural nitrogen-absorption efficiency, BY: biomass yield, CAT: catalase, DTF: days to 50% flowering, FAA: free amino acid, GY: grain yield, GW: grain weight, GD: grain density, HI: harvest index, NGP: number of grains per panicle, NGOC: NADH-glutamate synthetase content, NC: nitrogen content, NUE: nitrogen use efficiency, NR: nitrate reductase, PL: panicle length, PH: plant height, RDW, root dry weight; RL, root length, NR: number of roots, RT, root thickness, SPAD: chlorophyll content, POD: peroxidase, PNP: panicle number per plant, PFP: partial factor productivity, PS: photosynthetic rate, SF: spikelet fertility, SDW: shoot dry weight, SP: soluble protein, TN: tiller number, TDB: total dry biomass, Chr: chromosome, Mb: megabase, cM: centimorgan, CI: confidence interval.

**FIGURE 2:**
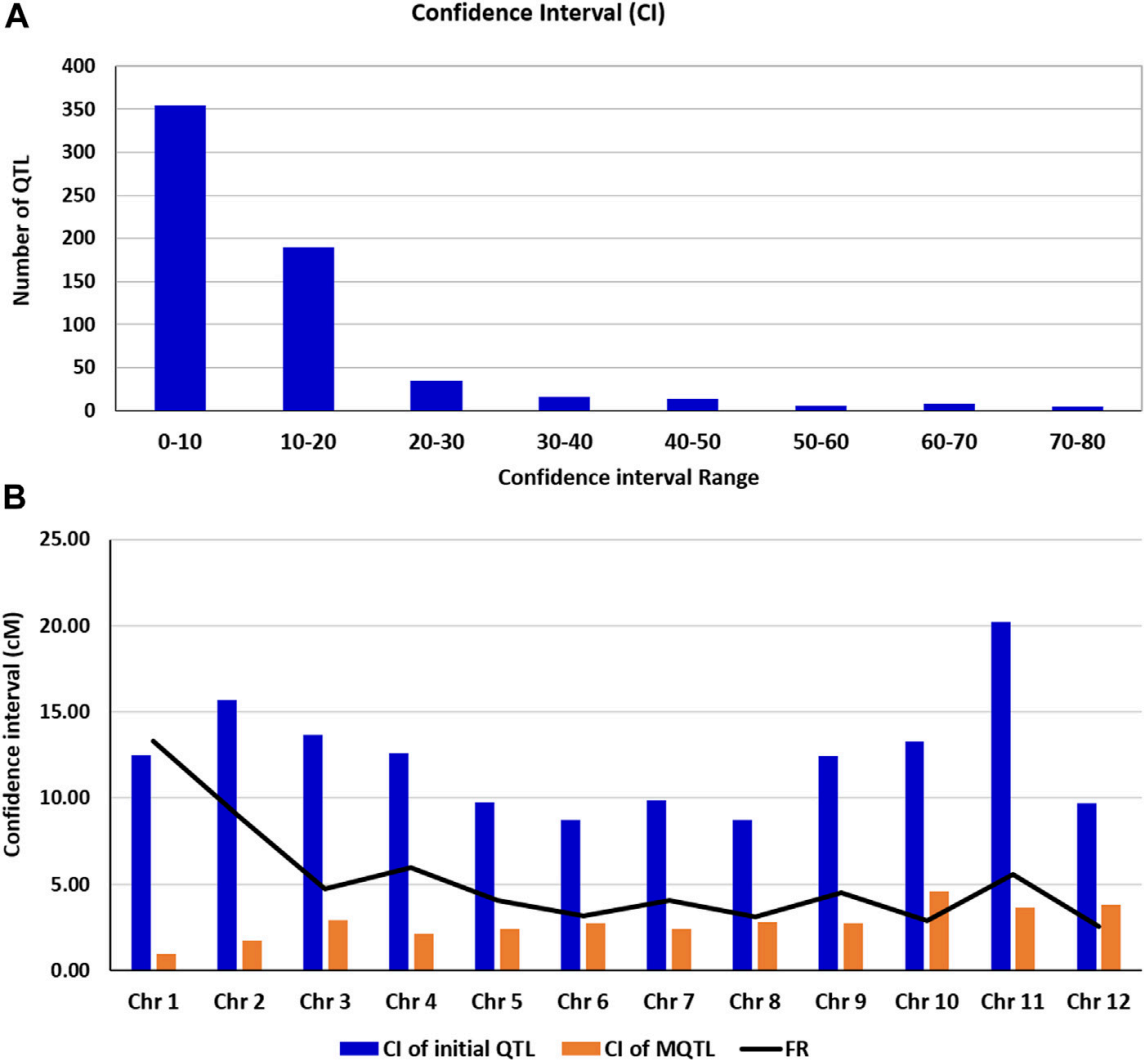
**(A)** Frequencies of QTL with different sizes of confidence interval (in cM) **(B)** (b) Comparison of confidence intervals (CIs) of the QTL and those of meta-QTL (MQTL), showing the fold level of reduction (FR) in the size of CI.

The phenotypic variance of the detected MQTL ranged from 8.14 to 21.16, and the LOD ranged from 3.29 to 9.47. The confidence interval of the MQTLs, on an average, was reduced 5.24-fold relative to the confidence intervals of the QTLs used in the present study. The average confidence interval of the QTL used in the present study ranged from 8.72 to 20.20 cM with an average confidence interval of 12.26 cM. The frequencies of QTL with different sizes of confidence interval (in cM) are presented in Figure 2A. The average confidence interval of the MQTLs detected in the present study varied from 0.94 to 4.57 cM with an average confidence interval of 2.73. The average reduction in the size of the confidence interval for individual MQTLs was as high as 13.3-fold for chromosome 1 and 8.95-fold for chromosome 2, and as low as 2.9-fold for chromosome 10 and 2.55-fold for chromosome 12 (Figure 2B).

Each of the individual MQTL differed for the number of traits controlling the MQTL (Table 3). The association of MQTL with traits varied from a single trait for MQTL6.1 and MQTL12.6 to 21 traits for MQTL1.1. The 20 MQTLs out of 76 detected MQTLs were reported to be associated with more than 10 traits (Table 3). The 55 MQTLs were reported to be associated with nitrogen use efficiency–related traits, root traits, and grain yield/yield-related traits (Table 3). The major colocation of genomic regions associated with root traits improving the nitrogen use efficiency and grain yield/related traits was observed on chr 2, 3, 4, 5, 6, and 10 (Table 3). The MQTL analysis for the 630 QTLs associated with the grain yield and yield-related traits indicates the presence of MQTL for yield and related traits in the same genomic region (Supplementary Table S1). A total of 11 MQTLs (MQTLYRT1.3 on chr 1, MQTLYRT2.1 and MQTLYRT2.4 on chr 2, MQTLYRT3.4 and MQTLYRT3.5 on chr 3, MQTLYRT4.3 on chr 4, MQTLYRT5.2 on chr 5, MQTLYRT6.5 on chr 6, MQTLYRT7.2 on chr 7, and MQTLYRT8.2 and MQTLYRT8.4 on chr 8) were observed to be associated with more than 6 yield and yield-related traits (Supplementary Table S1). The 13 MQTLs (MQTLN&R1.1 on chr 1; MQTLN&R2.4 on chr 2; MQTLN&R3.1 and MQTLN&R3.2 on chr 3; MQTLN&R4.3, MQTLN&R4.4, and MQTLN&R4.5 on chr 4; MQTLN&R5.2 and MQTLN&R5.3 on chr 5; MQTLN&R6.1 and MQTLN&R6.9 on chr 6; MQTLN&R7.4 on chr 7; and MQTLN&R11.4 on chr 11) showed association with the root traits improving nitrogen use efficiency in rice (Supplementary Table S2). The 15 MQTLs were solely associated with nitrogen use efficiency in rice (Supplementary Table S2).

### Identification of Candidate Genes and Orthologues

The 76 MQTLs reported in the present study were used for further selection of some of the promising MQTLs using the criteria; average phenotypic variance >8%, average LOD >4, and the involvement of ≥10 initial QTLs within the MQTL. This screening of MQTL resulted in the selection of 42 promising MQTLs (Supplementary Table S3), which was further used for the identification of candidate genes and orthologues in other crops such as wheat, barley, and maize.

A total of 2665 genes were present in the genomic region constituting the 42 promising MQTLs (Supplementary Table S4). A total of 158 candidate genes associated with plant growth and development, amino acid biosynthesis, nitrogen assimilation and transport, and stress resistance/tolerance were chosen (Supplementary Table S5) to identify the orthologues in barley, maize, and wheat. Out of the 158 candidate genes, 39 candidate genes showed no syntenic relationship with any of the three genomes i.e., barley, maize, and wheat. The 109, 104, and 94 of these 158 rice candidate genes could be utilized to identify the 376 wheat orthologues, 149 maize orthologues, and 109 barley orthologues, respectively, in the MQTL region (Supplementary Table S6).

### Collinearity Within the Rice Genome and Synteny With Other Genomes

**FIGURE 3:**
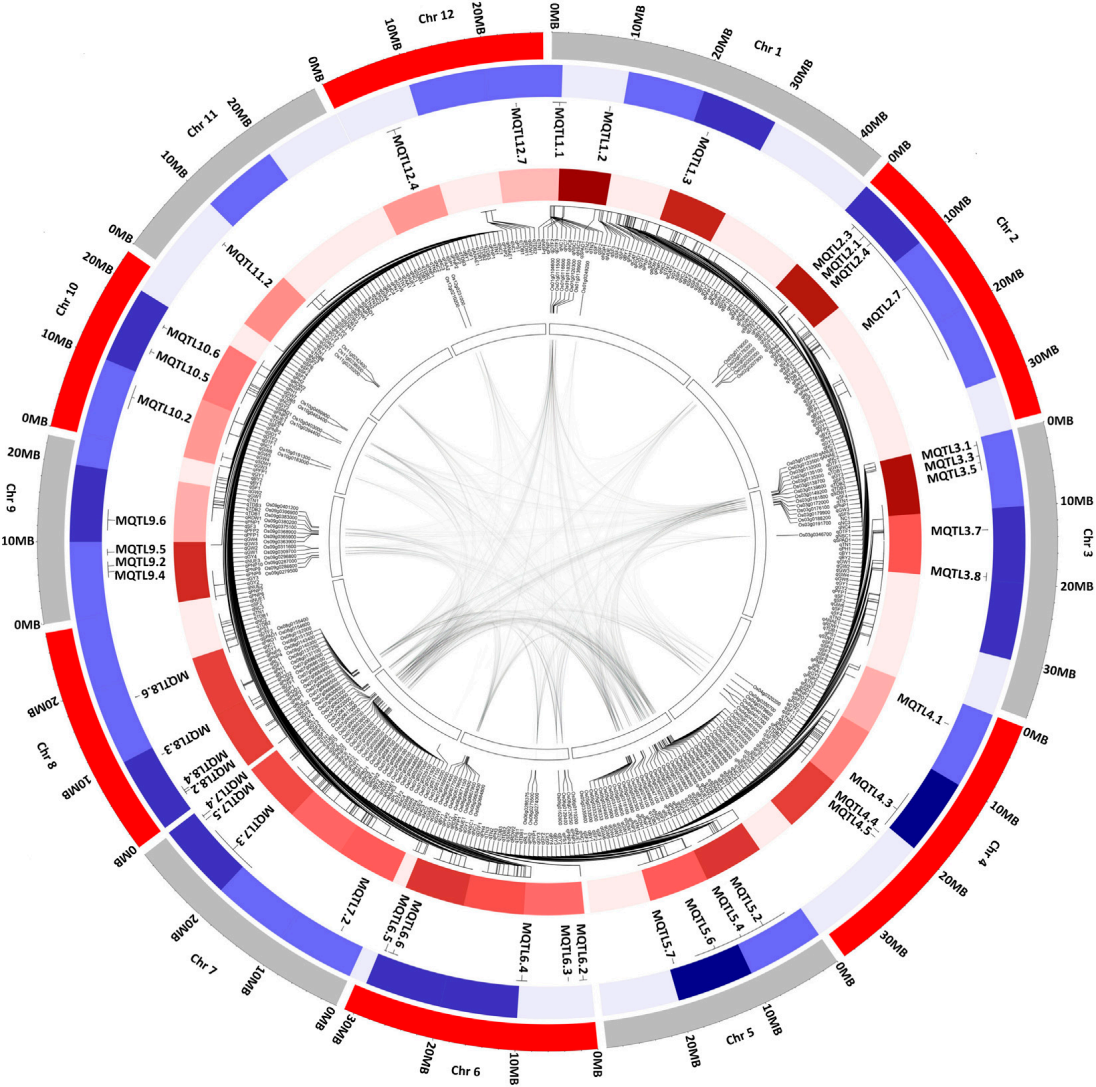
Schematic representation of the distribution pattern of identified MQTL, QTL, and candidate genes on rice chromosomes. From the center of the plot moving to the outer circle (1) The innermost circle representing the collinearity within the rice genome (2) Candidate genes identified in the major MQTL region (3)(query) QTL density in the MQTL region (4) MQTLs associated with nitrogen use efficiency and related traits (5) Outermost circle represents the rice genome in MB. The color density indicates the number of MQTL detected and number of QTLs present in the MQTL region. The denser color indicates a greater number of MQTL or QTL within the MQTL region.

The investigation of collinear genomic regions within the rice genome resulted in the identification of duplicated regions containing the MQTLs associated with the same traits. The candidate genes underlying MQTL7.4 and MQTL9.6 were reported to be associated with the cellular response to nitrate, and MQTL1.2 and MQTL5.2, with nutrient reservoir activity (Supplementary Table S6). The candidate genes in the genomic region of MQTLs on chr 3, 5, 6, 7, 8, 10, 11, and 12 were observed to be associated with the transmembrane transport activity (Supplementary Table S6). The collinearity in the genomic region on chr 1,5; chr 2,3; chr 3,6; chr 3, 7; chr 4, 6; chr 5, 6; chr 5, 7; chr 6,9; and chr 7,9 was observed (Supplementary Table S6). The MQTLs associated with the nitrogen content, root traits, and grain yield such as MQTL1.1 and MQTL1.3 on chr 1; MQTL3.3 and MQTL3.5 on chr 3; MQTL4.3 and MQTL4.5 on chr 4; MQTL5.5 on chr 5; MQTL6.2 and MQTL6.3 on chr 6; MQTL7.2 on chr 7; MQTL8.6 on chr 8; MQTL9.4, MQTL9.5, and MQTL9.6 on chr 9; and MQTL11.4 on chr 11 were co-located in the rice genome duplicated regions (Figure 3). The already reported genes have been investigated in the MQTL region using the Q-TARO database. The MQTL1.1 comprises the *OsDET1* gene associated with photosynthetic capacity, *sui1* gene with plant height, and *AIP1* gene with root hair development; MQTL2.7 comprises the *GW2* gene associated with grain weight and size and *OsNAR2.1* gene associated with nitrogen uptake; MQTL3.5 comprises the *OsMDP1* gene associated with root elongation; MQTL4.6 comprises the *OsAMT1;1* gene associated with ammonium uptake; and MQTL12.6 comprises the *kch1* gene associated with coleoptile elongation (Supplementary Table S7).

**FIGURE 4:**
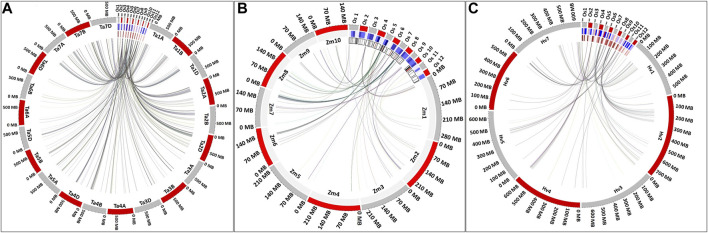
Syntenic relationship of rice candidate genes present in the MQTL region with the **(A)** wheat, **(B)** maize, and **(C)** barley genome.

The syntenic relationship was observed for rice candidate genes present in the MQTL region with the wheat, maize, and barley genome (Supplementary Table S8). The comparison of rice genome with wheat (Figure 4A), maize (Figure 4B), and barley (Figure 4C) genomes suggested that most of the orthologues were retained during evolution.

### Checking the Efficacy of MQTL

**FIGURE 5:**
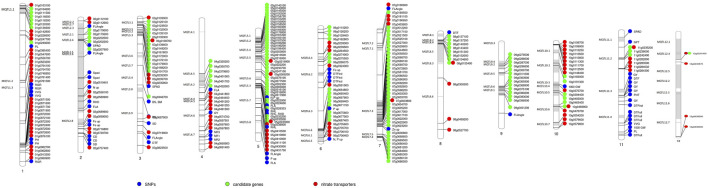
Schematic representation of the distribution of previously reported single nucleotide polymorphism (SNP) associated with traits of interest, candidate genes reported in the present study, and the nitrate transporter along the 7 chromosomes of rice. The chromosome map showing MQTL identified in the present study and their co-location with the previously reported single nucleotide polymorphism (SNP) associated with different nitrogen use efficiency (NUE)–related trait, root traits, yield, and yield-related traits (Sandhu et al., 2015; Sandhu et al., 2019; Subedi et al., 2019), candidate genes reported in the present study, and the nitrate transporter reported in rice. The numbers below each rice chromosome indicate chromosome numbers.

**FIGURE 6:**
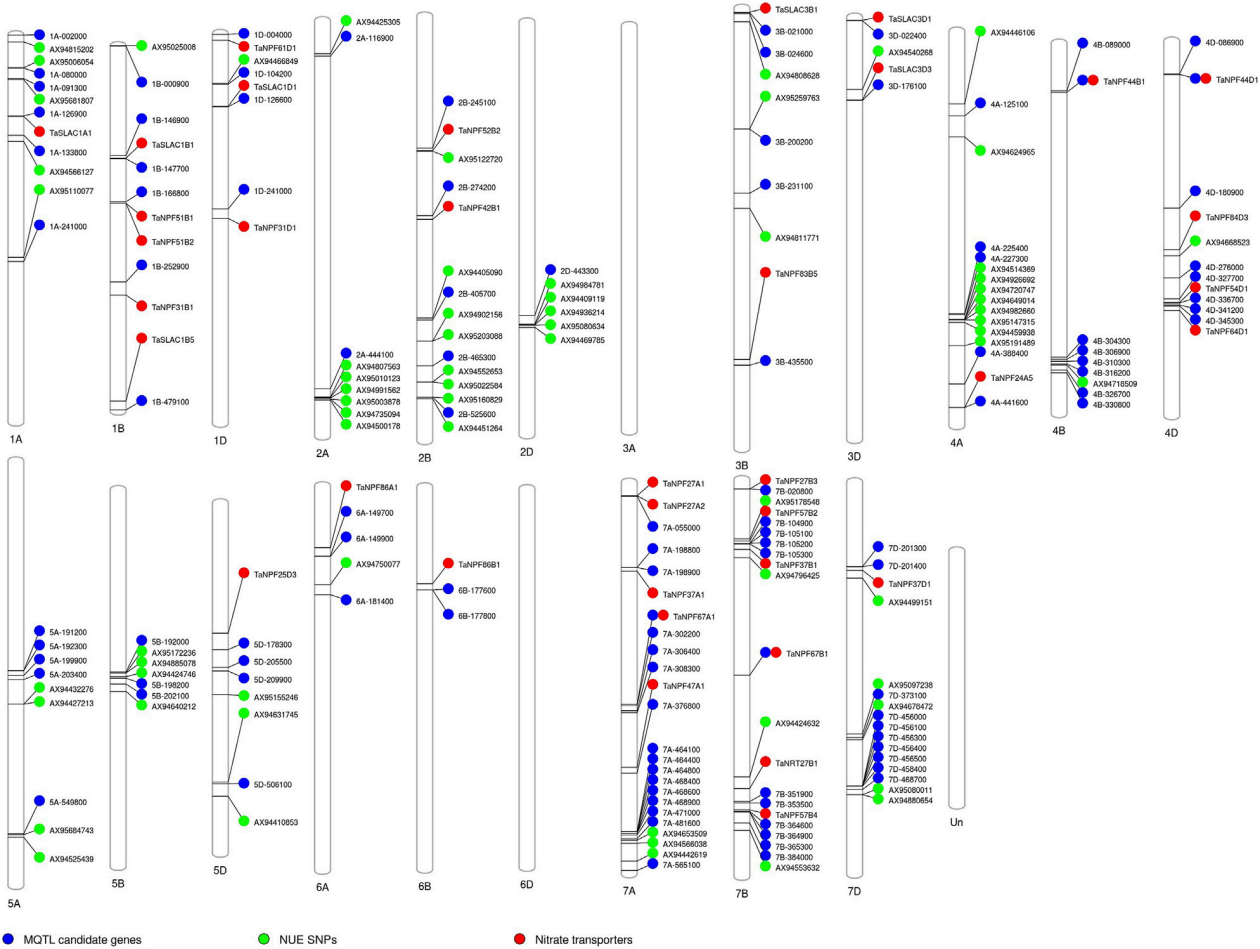
Schematic representation of the distribution of previously reported single nucleotide polymorphism (SNP) associated with traits of interest, candidate genes reported in the present study, and the nitrate transporter along the 21 chromosomes of wheat. The chromosome map showing the candidate genes reported in the present study in the MQTL gene based on the synteny and their co-location with the previously reported single nucleotide polymorphism (SNP) associated with different nitrogen use efficiency (NUE)–related trait, root traits, yield, and yield-related traits (Sandhu et al., 2021), and the nitrate transporter reported in wheat (Kumar et al., 2021). The numbers below each rice chromosome indicate chromosome numbers.

To check the efficacy of identified MQTL and candidate genes underlying MQTL, a validation panel of the marker-trait association identified in our previous study (Sandhu et al., 2015; Sandhu et al., 2019; Subedi et al., 2019) in rice and wheat (Sandhu et al., 2021) and nitrate transporters reported in rice was made. The identification of previously identified marker-trait associations associated with the nitrogen use efficiency–related traits in rice and nitrate transporter genes in close proximity to the MQTL and candidate genes reported in the present study indicated the robustness of the reported MQTL. The marker-trait association associated with the root traits (root hair length and root hair density) improving nutrient uptake (nitrogen and phosphorus) was reported to be collocated or in close proximity to the nitrogen transporter genes and candidate genes identified in the present study on chr 2, 5, and 6 (Figure 5). The marker-trait associations associated with grain yield/yield-related traits and plant morphological traits reported in our previous studies were present in the MQTL region reported in the present study. Similarly, the wheat orthologues were present in close proximity to the marker-trait association and nitrate transporter genes identified in our previous study (Kumar et al., 2021; Sandhu et al., 2021) (Figure 6). The identified 20 candidate genes in wheat are nitrogen transporters.

### Expression Profiles of Candidate Genes

**FIGURE 7:**
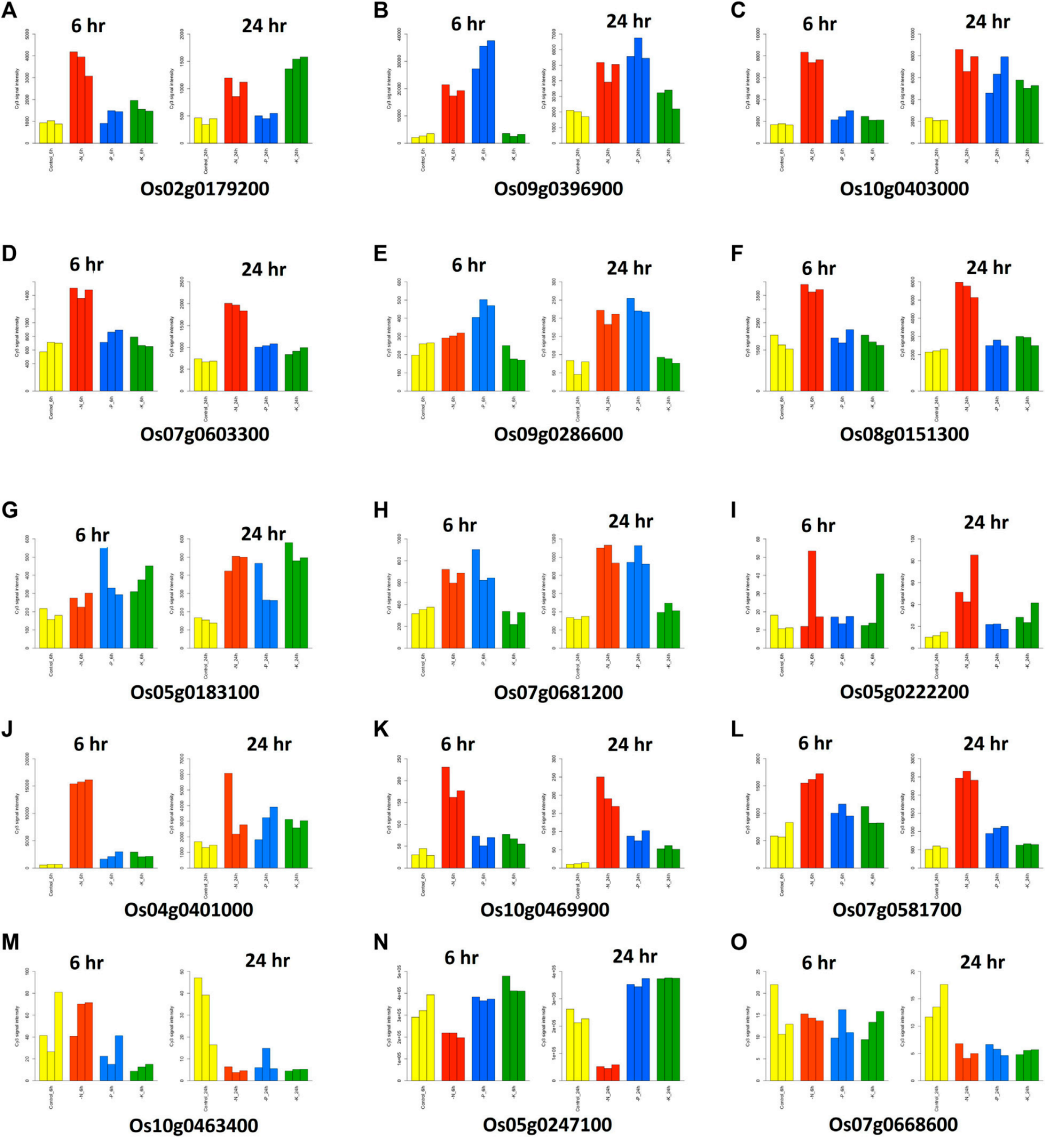
Graphical representation of the significant changes in expression values of the candidate genes in response to nitrogen deficiencies in roots.

To identify the target genes for NUE, we analyzed the expression data of all 158 candidate genes identified in this study. We studied the response of candidate genes against nitrogen deficiency in the microarray-based expression dataset from the RiceXPro database. Of all the 158 candidate genes, 15 genes were selected which showed significant changes in expression values in response to nitrogen deficiencies in roots (Table 4). Twelve out of 15 genes showed upregulation in response to nitrogen deficiency, while three genes were downregulated (Figure 7). Eight genes out of twelve were upregulated in both 6 and 24 h after treatment in comparison to control. These genes consisted of glutamine amidotransferase, *OsVIT2*, *PLA1*, *GL7*, ERF transcription factor (*Sub1C*), *CEF1*, HD zip TF (*Oshox14*), and TGF beta receptor (Table 4). Three genes (*OsWRKY67*, acid phosphatase, and *OsABCG12*) showed a similar expression to control at 6 h after treatment, but their expression increased at 24 h. Meanwhile, one gene showed more expression at 6 h after treatment, but its expression was similar to control at 24 h after treatment (Figure 7). Three genes showed similar expressions at 6 h after treatment, but their expression decreased significantly after 24 h in comparison to control (Figure 7). These genes consisted of *EF1*(B type response regulator), *DIP3* (glycosyl hydrolase family 18), and *OsGZF1* (Zn finger CCCH domain containing protein) (Table 4). There were 5 genes that had multiple transcripts or splice variants, but no variation was detected between transcripts of the same gene.

**TABLE 4:** Selected 15 candidate genes showing response to nitrogen treatment.

MQTL	Gene stable ID	Gene description	Gene name
*MQTL2.1*	*Os02g0179200*	Glutamine amidotransferase class-I domain containing protein	-
*MQTL4.3*	*Os04g0401000*	Proline-rich protein, Blast resistance	*PI21*
*MQTL5.2*	*Os05g0183100*	Similar to WRKY transcription factor 16 (Fragment)	*OsWRKY67*
*MQTL5.2*	*Os05g0222200*	ABC transporter-like domain containing protein	*OsABCG12*
*MQTL5.2*	*Os05g0247100*	Similar to Glycosyl hydrolases family 18	*DIP3*
*MQTL7.3*	*Os07g0581700*	Homeodomain-leucine zipper (HD-Zip) transcription factor	*Oshox14*
*MQTL7.3*	*Os07g0603300*	TON1 RECRUIT MOTIF (TRM)-containing protein	*GL7*
*MQTL7.5*	*Os07g0668600*	Zinc finger, CCCH-type domain containing protein	*OsGZF1*
*MQTL7.4*	*Os07g0681200*	Vegetative storage protein/acid phosphatase domain containing protein	-
*MQTL8.2*	*Os08g0151300*	R2R3-MYB transcription factor	*CEF1*
*MQTL9.2*	*Os09g0286600*	Pathogenesis-related transcriptional factor and ERF domain containing protein	*Sub1C*
*MQTL9.6*	*Os09g0396900*	Protein of unknown function DUF125, transmembrane family protein	*OsVIT2*
*MQTL10.5*	*Os10g0403000*	Cytochrome P450 protein, CYP78A11	*PLA1*
*MQTL10.6*	*Os10g0463400*	B-type response regulator	*EF1*
*MQTL10.6*	*Os10g0469900*	TGF-beta receptor, type I/II extracellular region family protein	-

## Discussion

Among all the plant nutrients basic for crop development, N is the nutrient limiting the crop productivity. Overabundance use of nitrogenous fertilizers creates imbalance in ecosystem function and services (Fowler et al., 2013). There is a strong requirement to enhance nitrogen use efficiency for the sustainable agriculture production (Paul et al., 2014). The identification of genomic regions associated with traits of interest using molecular markers is an accurate and useful approach in the marker-assisted breeding program (Ashikari and Matsuoka, 2006; Price, 2006). The complex nature of the QTL and their interaction with other QTLs, genetic background, and environment are some of the important constraints in identifying their precise location. The identification of a major effect and consistent QTL across different genetic backgrounds and environments is an important requirement for the precise use of the identified genomic regions in marker-assisted selection.

The meta-analysis of the genomic regions reported in different studies helps in identification of most accurate and confined genomic regions to be further used in the marker-assisted introgression program. The trait NUE is not a biological measure by itself; it is a complex derivative of biological measures such as the grain yield/related traits and nitrogen response. A number of studies reported QTL associated with NUE in rice (Anis et al., 2019; Jewel et al., 2019; Mahender et al., 2019; Zhang et al., 2019). To the best of our knowledge, many QTLs involved in the NUE were reported separately, but a comprehensive listing and analysis of the genomic region associated with the grain yield and nitrogen use efficiency were not available. In the present study, the meta-QTL analysis was performed to identify the consistent and major effect QTL associated with nitrogen use efficiency in rice.

The present study explored 1,330 QTLs associated with NUE and related traits in rice and identified a total of 76 MQTLs, suggesting the power of MQTL analysis in narrowing down the genomic regions controlling the different traits of interest (Khahani et al., 2020). The number of available QTLs that we listed here was 1,330 QTLs of which 915 major QTLs were used for the identification of as many as 76 MQTLs, suggesting that to the best of our knowledge, the present study is so far the most comprehensive study for the identification of NUE-associated MQTLs in rice. The first attempt to map the genomic region associated with NUE and related traits was initiated at IRRI. The distribution of the QTL on different rice chromosomes with the highest number of QTL on chr 1 and 3 was similar to that of the previous reports (Swamy et al., 2011; Swamy and Sarla, 2011; Khahani et al., 2020). Some of the MQTLs identified in the present study constituted as high as 47 initial QTLs per MQTL (MQTL1.1), indicating the robustness of the detected MQTL. The consensus map developed in the present study is much informative relative to those prepared and used in the earlier studies for the identification of MQTL associated with the traits (Courtois et al., 2009; Swamy et al., 2011; Khahani et al., 2021; Kumari et al., 2021). The fold reduction in the size of the confidence interval of the MQTL detected in the present study is in contrast to those reported in the previous studies (Courtois et al., 2009; Danan et al., 2011). The reduction in the confidence interval allows for the exploration of most promising and less number of candidate genes per MQTL.

Here, we ran the MQTL analysis separately for the grain yield/yield-related traits and nitrogen use efficiency–related traits also to identify the major and consistent genomic regions associated solely with the trait. However, it is more effective and robust to pool different correlated traits measured in the same population (Goffinet and Gerber, 2000), assuming that a number of traits studied are pleiotropically related. The analysis of pooled correlated traits may be more powerful than the analysis of an individual trait ensuring better coverage of the genome than the single trait analysis. This enabled us to identify the stable QTL and “hotspots” for the NUE-related traits in rice across different genetic and environmental backgrounds to be further used in the marker-assisted introgression program.

Our approach of using all correlated traits altogether in the MQTL analysis in the present study was more robust, not only because we used all correlated traits but also we targeted only the genomic region (phenotypic variance >8) associated with NUE-related traits to shortlist the target genes. The co-location of root traits improving NUE and yield/yield-related traits in the duplicated region of the rice genome might be useful in identification of promising candidate genes controlling the traits of interest. The MQTLs reported in the present study showed a positive effect on the grain yield/yield-related traits, root traits improving nutrient uptake, NUE, and related traits. The presence of candidate genes *Os02g0207900* (MQTL2.4), *Os04g0379600* (MQTL4.3), *Os06g0286375* (MQTL6.4), and Os08g0155400 (MQTL8.2) encoded nitrate transporters, suggesting the efficacy of the detected MQTL. The MQTL on chr 1, 4, and 8 reported as a hotspot with 21, 12, and 12 yield/yield-related and NUE-related traits, respectively. The MQTL on chr 2 and 6 constituting genes associated with the nitrate/ammonium uptake/content (*OsNAR2.1, OsAMT1;1, OsPTR9*, *OsAAT49*) (Yan et al., 2011; Lu et al., 2012; Fang et al., 2013; Ranathunge et al., 2014). The identification of genes encoding the nitrate transporter may be very useful to target for improving nitrogen use efficiency under direct seeded cultivation conditions where the soil conditions are dry and nitrate is the major source of nitrogen. Various other reported genes and candidate genes associated with the floral organ development, grain size, grain weight, fertility, and root development have been found in the MQTL region detected in the present study (Supplementary Tables S7, S8, S9).

The rice genomic region subtending the MQTL4.3 (*Os04g0379600*) and MQTL8.2 (*Os08g0155400*) harbor the candidate genes encoding nitrate transporters; we identified the orthologue of these genes in wheat (*Os04g0379600: TraesCS2D02G279200, TraesCS2A02G280400, TraesCS2B02G297700; Os08g0155400: TraesCS7B02G201900, TraesCS7A02G301700, TraesCS7D02G297000*), maize (*Os04g0379600: Zm00001eb085850; Os08g0155400: Zm00001eb416090*), and barley (*Os08g0155400: HORVU7Hr1G071600*). The other genes associated with nitrogen uptake/transport/assimilation, amino acid synthesis/transport, protein synthesis/transport/phosphorylation in the MQTL region, and their orthologues in wheat, maize, and barley have been reported in the present study (Supplementary Tables S7, S8), providing a better understanding of candidate genes controlling NUE with a similar evolutionary background and conserved the function between different cereal crops. The root-specific expression of the candidate genes was identified in the MQTL region under the nitrogen-deficit condition, suggesting the role of root traits in improving NUE in plants. The identification of nitrate transporter genes and previously identified marker-trait associations in rice and wheat (Sandhu et al., 2015; Sandhu et al., 2019; Subedi et al., 2019; Kumar et al., 2021; Sandhu et al., 2021) in close proximity to the MQTL/candidate genes/their orthologues associated with the NUE-related traits in the present study validates the efficacy of the reported MQTL. The 76 MQTLs reported in the present study were used for a further selection of some of the promising MQTL using the criteria; average phenotypic variance >10%, average LOD >4, and the involvement of ≥10 initial QTLs within the MQTL. Furthermore, the MQTLs were screened on the basis of phenotypic variance, LOD, and number of QTL in the MQTL region and resulted in the selection of 42 promising MQTLs, which were further used for the identification of candidate genes and expression studies. Once validated, the identified 15 target genes for NUE in the MQTL present on chromosomes 2,4,5,7,8,9, and 10 (Table 4) may be targeted for identification of donors possessing candidate genes and for marker development for further deployment in the marker-assisted breeding program.

## Conclusions

NUE is a quantitative trait controlled by multiple genes and the co-localization of genomic regions associated with yield/yield-related traits and root traits improving NUE providing key candidate genes for the rice crop improvement. The MQTL analysis approach is used in the present study overcoming the limitation of QTL mapping while felicitating identification of robust markers and fine-mapped genomic regions to be further used in the marker-assisted introgression program. The present study identified 76 MQTLs associated with NUE and related traits in rice. The study also verified the evolutionary relationship of cereal crops through the mining of orthologues using the ortho-MQTL approach. The results reported in the present study will be applicable to improve the selection for the yield/yield-related traits and root traits improving nitrogen use efficiency in rice-breeding programs. The detailed crosstalk between the genome and proteome and the validation of identified putative candidate genes in the MQTL region through gene expression and gene editing studies may lay down the foundation to improve the nitrogen use efficiency of cereal crops.

## Data Availability

The original contributions presented in the study are included in the article/Supplementary Material, further inquiries can be directed to the corresponding author.

## Supplementary Material

1000 GW: 1,000 grain weight, BS: bending strength, BM: bending moment, CD: culm diameter, DTF: days to 50% flowering, DTFirst: days to first emergence, DTFull: days to full emergence, Fe up: iron uptake, FLA: flag leaf area, FLAngle: flag leaf angle, GY: grain yield, NPT: number of productive tillers, NR: nodal root number, N up: nitrogen uptake, P up: phosphorus uptake, RGR: relative growth rate, RHL: root hair length, RHD: root hair density, PL: panicle length, PH: plant height, SPAD: chlorophyll content, SD: stem diameter, VVG: vegetative vigor, Zn up: zinc uptake.
